# Pathological, microbiological, and molecular characterization of uterine samples in culled sows from farms with a history of reproductive failure in Brazil

**DOI:** 10.1007/s11259-026-11435-6

**Published:** 2026-07-31

**Authors:** Jean Carlo Olivo Menegatt, Anderson Hentz Gris, Fernanda Felicetti Perosa, Adriano Tony Ramos, Francielli Cordeiro Zimermann, Carolina Reck, Álvaro Menin

**Affiliations:** 1 Laboratório de Patologia Veterinária, Universidade Federal de Santa Catarina - Campus Curitibanos, Curitibanos, Brazil; 2 Setor de Patologia Veterinária, Faculdade de Veterinária, Universidade Federal do Rio Grande do Sul, Porto Alegre, Brazil; 3 Department of Veterinary Diagnostic and Production Animal Medicine, Iowa State University, Iowa, United States of America; 4 Instituto de Pesquisa e Diagnóstico Veterinário, Vertà Laboratórios, Santa Catarina, Brazil; 5 Departamento de Biociências e Saúde Única, Universidade Federal de Santa Catarina, Santa Catarina, Brazil

**Keywords:** Bacteriology, Chronic endometritis, Histopathology, Swine, Uterus

## Abstract

Reproductive failure in swine herds is an important cause of production losses and increased sow culling. While many diagnostic efforts focus on abortion-derived samples, the evaluation of reproductive tracts from culled sows may provide additional insights. This study aimed to characterize the histopathological, microbiological, and molecular alterations focused on uterine samples of sows from three Brazilian farms with a history of reproductive failures. A total of 207 reproductive tracts from culled sows were collected at slaughter and examined for gross, histological lesions, and bacteriological analysis. Uterine samples were also analyzed via PCR for key pathogens linked to reproductive failure, including viruses (PCV-2, PCV-3, PPV), bacteria (*Brucella suis*, *Leptospira* spp., *Listeria* spp., *Mycoplasma suis*, *Erysipelothrix rhusiopathiae*), and protozoa (*Toxoplasma gondii*). Twenty-four out of 207 samples (11.6%) were positive for *Erysipelothrix rhusiopathiae*, 21/207 (10.1%) for PCV-2, 14/207 (6.8%) for porcine parvovirus (PPV), 13/207 (6.3%) for PCV-3, and 10/207 (4.8%) for *Leptospira interrogans*. Fifteen samples were concurrently positive for two (12/15) or three (3/15) of these infectious agents. Endometritis was observed in 21.74% (45/207) of the samples. The predominant histological patterns of endometritis were mixed (44.5%; 20/45), followed by chronic (26.7%; 12/45), mononuclear (22.2%; 10/45), suppurative/fibrinosuppurative (4.4%; 2/45), and pyogranulomatous (2.2%; 1/45). Findings of chronic endometritis resembling those described in mares with reproductive failures included mainly peri-glandular fibrosis and glandular ectasia. Bacterial culture yielded positive results in 57.7% (26/45) exclusively from endometritis cases, with *Escherichia coli*, *Staphylococcus aureus*, and *Streptococcus suis* being the most frequently isolated pathogens. Ovarian pathology accounted for twenty-four cases (24/207; 11.6%), including stage of anestrus (13/24) and ovarian follicular cysts (11/24). As individual reproductive histories and culling reasons of the sows were unavailable, these findings should be interpreted descriptively rather than as causal evidence of reproductive failure. In this context, the results represent a herd-level screening of circulating pathogens and may guide future farm-specific investigations.

**Supplementary Information:**

The online version contains supplementary material available at https://doi.org/10.1007/s11259-026-11435-6.

## Introduction

Reproductive failure is a significant cause of production losses and culling in commercial sow herds (de Jong et al. 2014; Althouse et al. 2019). Studies report that 13–49% of sows were culled due to reproductive failures, with some studies reporting as high as 30–50%, depending on the country (Engblom et al. 2008; de Jong et al. 2014; Althouse et al. 2019; Koketsu and Iida 2020). In Brazil, commercial herds typically have an expected farrowing rate of around 90%, meaning that about 10% of sows do not reach parturition. Abortion is one of the most significant consequences of reproductive failure, usually accounting for about 1.5–2% of these cases (Salogni et al. 2016; Maes et al. 2023). However, establishing a definitive cause remains elusive. This difficulty arises due to the complexities of performing and interpreting serological tests and the lack of correlation between infectious agents identified in the field and their pathological, serological, and molecular findings (Pescador et al. 2010; Menin et al. 2019; Maes et al. 2023).

Part of the challenge in diagnosing reproductive failure is also linked to its multiple underlying causes. About 30–40% of abortion cases are related to infectious agents, while the remaining 60–70% are non-infectious and often difficult to identify, especially through pathological analysis (Pescador et al. 2010). Some non-infectious causes include genetic, toxic, nutritional, climate, and environmental factors (Pescador et al. 2010; Nikkilä et al. 2013; Iida and Koketsu 2015). Additionally, many other factors contribute to reproductive losses, such as repeated estrous cycles, semen quality, management practices, underlying diseases or comorbidities (e.g., foot lesions), and ovarian changes (Castagna et al. 2004; Bertoldo et al. 2012; Pozzi and Alborali 2012; Althouse et al. 2019).

Most studies on reproductive losses in swine focus on abortion, aiming to identify causes through analyses of fetuses and/or placenta (Pescador et al. 2010; Salogni et al. 2016; Menin et al. 2019; Althouse et al. 2019; Maes et al. 2023). While a thorough examination of the sow reproductive tract at slaughterhouses can reveal uterine lesions linked to reproductive failure (Dalin et al. 1997; Heinonen et al. 1998; Tummaruk et al. 2009; de Jong et al. 2014), histopathological analysis may be more sensitive for detecting lesions formed due to conditions such as endometritis (de Jong et al. 2014). To date, there is a lack of studies exploring a wide range of reproductive pathogens in uterine samples from sows with reproductive issues. Therefore, this study aims to conduct microbiological, molecular, and histopathological analyses of uterine samples from slaughtered sows on Brazilian commercial farms with a history of reproductive failure.

## Materials and methods

### Study population

This study was conducted between January and July 2022 on three farms in Santa Catarina, Brazil, each with a documented history of reproductive failure. The herd sizes were approximately 500 sows (Farm 1), 4,000 sows (Farm 2), and 1,500 sows (Farm 3). These herds exhibited annual sow replacement rates of 40% to 45%, monthly abortion rates of 2.5% to 4%, and farrowing rates between 84% and 86%. Sampled sows were culled for unspecified reasons, and individual reproductive data, such as parity, interval since last farrowing or weaning, and number of services, were not accessed. Convenience samples of the entire reproductive tract (ovaries, uterine tubes, and uterus) were collected at slaughter, refrigerated between 2 and 8 °C, and transported to VERTA Laboratories within 12–24 h for analysis. During each sampling event at the slaughterhouse, reproductive tracts from all culled sows originating from the three farms on that occasion were collected, with no prior selection of animals. Each entire reproductive tract (sample) corresponded to one sampled sow. From each farm, two or three batches of culled sows were investigated until they represented one month of the annually culled sows. Gilts were not included in the study.

### Gross examination

Samples were evaluated macroscopically to detect lesions in the ovaries, uterine tubes, and uterus by two pathologists simultaneously (JCOM and AHG). The entire length of the uterus was palpated and incised to search for lesions or pregnancy status. Uterine gross lesions were recorded only if signs of infection or conditions that may correlate with reproductive failure (such as macerated fetuses or uterine exudate) were evident. The findings were documented as present or absent by the consensus of both pathologists.

Reproductive status was evaluated based on ovarian characteristics (Heinonen et al. 1998). Briefly, the animals were classified as anestrous sows if the ovaries were inactive (absence of corpora lutea or follicles less than 5 mm in diameter) or cycling sows if the ovaries had shown activity (estrous: follicles between 5 and 15 mm; diestrus: presence of corpora lutea). Ovarian follicular cysts were considered when follicles were larger than 15 mm in the ovarian parenchyma. Cysts located in the mesosalpinx area, adjacent to the uterine tubes, were classified as paratubal cysts.

### Bacteriological examination

For qualitative bacteriological examination (presence/absence), the external surface of the uterus was decontaminated with 70% ethanol. The uterus was then aseptically opened using a sterile scalpel along the longitudinal axis from the uterine horns toward the cervix, and a long sterile swab was used to collect from the intrauterine lumen at the anatomical junction between the cervix and the bifurcation of the two uterine horns. Immediately after collection, samples were refrigerated at 2–4 °C and processed within 12 h.

The intrauterine swab was subjected to bacterial culture and phenotypic characterization following standard microbiological procedures (Quinn et al. 2012). Enriched non-selective media, including 5% sheep blood agar and GC agar supplemented according to the manufacturer’s recommendations, were inoculated and incubated under aerobic and capnophilic conditions (aerobiosis with 5% CO₂) to support the growth of fastidious organisms. In parallel, selective media (MacConkey agar) were incubated under aerobic conditions, whereas selective and differential media (SPS agar and anaerobic agar) were incubated under capnophilic (5% CO₂) and strictly anaerobic conditions to facilitate the recovery of specific bacterial groups.

Primary cultures were incubated at 35–37 °C and examined after 24, 48, and 72 h for bacterial growth. Colonies obtained were evaluated by Gram staining and subsequently subjected to conventional biochemical testing for phenotypic identification. These tests included assessment of spore formation, catalase and oxidase activity, carbohydrate fermentation with evaluation of acid and gas production, motility, and enzymatic or substrate degradation. The latter comprised methyl red and Voges–Proskauer reactions, Simmons citrate utilization, urease activity, indole production, nitrate reduction, gelatinase, DNase, lipase, lecithinase, and β-galactosidase (ONPG) tests. In addition, amino acid metabolism was assessed through decarboxylation or deamination reactions involving lysine, ornithine, arginine, and phenylalanine. When appropriate, complementary assays such as coagulase, CAMP test, bile esculin hydrolysis, and growth in 6.5% NaCl were performed to confirm the identity of the bacterial isolates.

### Histopathological examination

For histopathological analysis, tissue fragments up to 2 cm in diameter from both ovaries, uterine tubes, and the uterus were collected and fixed in a 10% formalin solution. The ovaries were longitudinally sectioned, and a region including both the ovarian cortex and medulla was collected. From the uterus, three samples were collected, representing both uterine horns and the cervix. Uterine horn fragments were collected bilaterally from a standardized site, extending up to 20 cm from the cervix. Fragments from the other horn regions presenting evidence or suspected lesions were also included when necessary, according to the consensus of both pathologists (JCOM and AHG). Tissue sections fixed in 10% formalin were routinely processed, embedded in paraffin, sectioned at 3 μm, and stained with hematoxylin and eosin (HE). Three to five slides from each sample were analyzed, comprising one fragment of each of the ovaries and uterine tubes, uterine horns, and cervix.

Histologically, the endometrium was examined to identify areas with inflammatory infiltrate. Five fields of 400x magnification (FN22) from the most affected areas (hot spots), defined as those with the highest density of inflammatory infiltrate in the lamina propria, were selected for evaluation. Field selection and assessment were performed by consensus between two veterinary pathologists (JCOM and FFP). The mean number of inflammatory cells counted across the five fields was used to classify endometritis. When inflammatory cells were absent to minimal (≤ 20 neutrophils or ≤ 40 lymphocytes, macrophages, plasma cells, and neutrophils), they were considered within the expected resident population, according to criteria adapted from Dalin et al. (2004) and Tummaruk et al. (2010). Cases with a mean count of ≥ 40 inflammatory cells were classified as endometritis. Endometritis cases were further categorized according to histomorphological patterns as follows: (1) Mononuclear (≥ 40 inflammatory cells: lymphocytes, macrophages, and plasma cells); (2) Mixed (≥ 40 inflammatory cells: mixed population of mononuclear cells and neutrophils); (3) Suppurative or fibrinosuppurative (≥ 40 inflammatory cells: predominance of neutrophils in addition to mucosa ulceration and fibrin deposition); (4) Pyogranulomatous (≥ 40 inflammatory cells: predominance of macrophages and neutrophils in addition to mild connective tissue proliferation); (5) Chronic endometritis (samples should present mononuclear endometritis in addition to fibrosis affecting > 5 gland branches in a 5.5 linear field). These lesions were evaluated based on previous studies on chronic endometritis from mares, where they corresponded to grade III endometritis (Snider et al. 2011; Barros and Masuda 2013; de Witt et al. 2023).

### Molecular examination

Under sterile conditions, a deep scraping of the uterine mucosa (endometrium) was performed to collect endometrial tissue from all samples using a sterile scalpel at the anatomical junction between the cervix and the bifurcation of the two uterine horns. Tissue fragments from this region were immediately collected, snap-frozen in liquid nitrogen, and transported under cryogenic conditions. Samples were subsequently stored at − 80 °C until further processing.

Genomic DNA was extracted from the endometrial scraping using a commercial extraction kit (MagMAX™ CORE Nucleic Acid Purification Kit, Thermo Fisher Scientific), according to the manufacturer’s instructions. DNA quality and concentration were assessed by fluorometric quantification (Fluoroquant, Loccus). The extracted DNA was subjected to polymerase chain reaction (PCR) assays performed on a QuantStudio™ 3 Real-Time PCR System (Thermo Fisher Applied Biosystems).

Molecular screening targeted infectious agents commonly associated with reproductive disorders in swine, including *Leptospira* spp. (Stoddard 2013; 242 bp), *Circovirus porcine2* (PCV-2; Olvera et al. 2004; 99 bp), *Circovirus porcine3* (PCV-3; Franzo et al. 2018; 113 bp), porcine parvovirus (PPV; McKillen et al. 2007; 99 bp), *Chlamydia* spp. (Pantchev et al. 2010; *Chlamydia suis*: 96 bp, *Chlamydia abortus*: 109 bp, *Chlamydia psittaci*: 76 bp, *Chlamydia pecorum*: 76 bp, *Chlamydia felis*: 78 bp, *Chlamydia caviae*: 84 bp), *Toxoplasma gondii* (Kompalic-Cristo et al. 2007; 115 bp), *Erysipelothrix rhusiopathiae* (Zhao et al. 2023; 217 bp), *Brucella suis* (Hänsel et al. 2015; 106 bp), *Listeria* spp. (Suo et al. 2010; 70 bp), and *Mycoplasma suis* (Hoelzle et al. 2007; 179 bp). Each PCR run included appropriate internal amplification controls, positive and negative controls, reaction controls, and evaluation of amplification efficiency to ensure analytical reliability. All molecular analyses were conducted in a laboratory accredited according to the ISO/IEC 17025:2017 international standard, supporting the accuracy and reliability of the generated results.

## Results

**Fig. 1:**
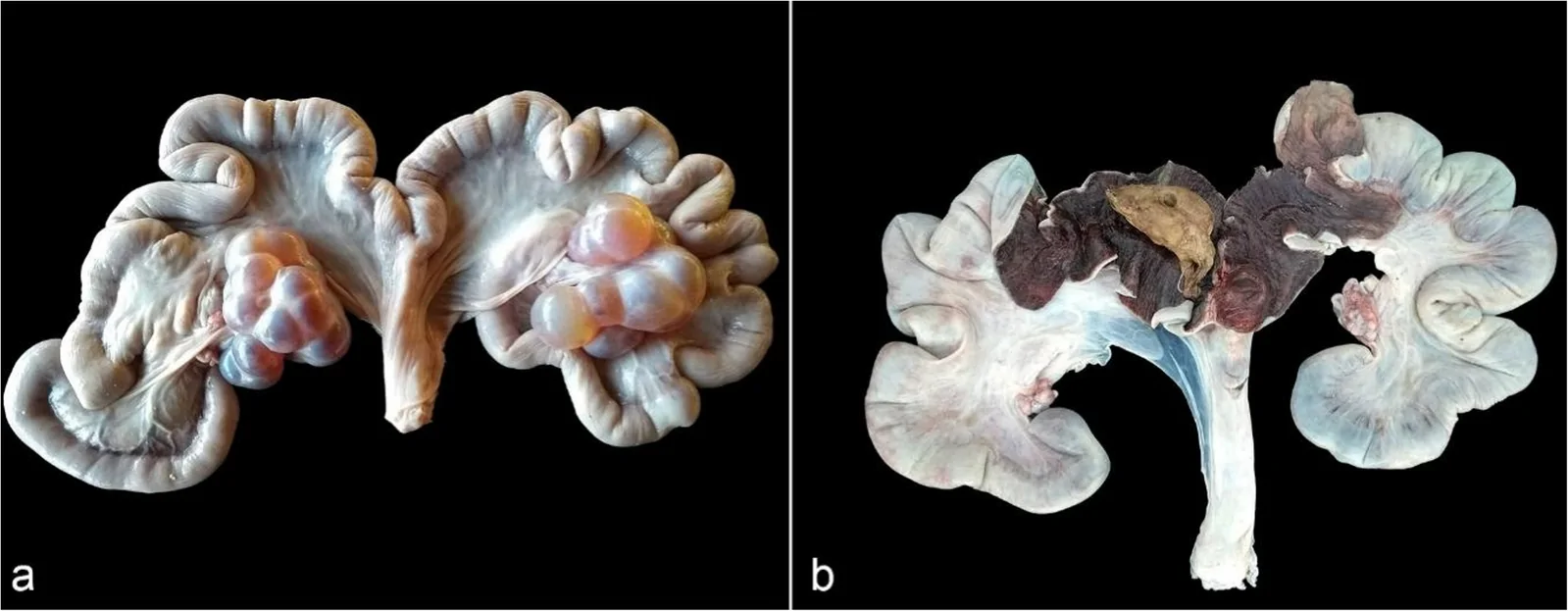
Macroscopic findings in reproductive tracts from culled sows with a history of reproductive failure. (**a)** Bilateral, numerous ovarian cysts measuring 15–30 mm in diameter. (**b) **A fetal skull within the uterine horn, suggestive of fetal maceration, co-exists with marked thickening of the endometrial mucosa

A total of 207 samples were collected (farm 1: 21 sows; farm 2: 145 sows; farm 3: 41 sows), and 49 samples presented macroscopic findings (23.7% [49/207]). The macroscopic findings for each farm are shown in Table 1. Ovarian alterations included anestrus and ovarian follicular cysts, which were single (8 samples) or numerous (3 samples) (Fig. 1a). Paratubal cysts were considered incidental findings, as no luminal obstructions caused by cysts were identified. In the endometrium, the main findings included the presence of exudate, macerated fetuses (Fig. 1b), and, in some cases, pregnancy (gestation). Additionally, three samples exhibited fibrinous inflammation extending from the peritoneum to the uterine serosa. The finding was associated with peritonitis in two sows and with an adjacent abscess in one sow, with no evidence of a primary uterine lesion in any of the three cases. No bacterial isolates were obtained from these samples. The more frequent reproductive status was found to be diestrus (84.54% [175/207]), followed by anestrus (6.28% [13/207]), estrus (5.31% [11/207]), and pregnancy (3.86% [8/207]).

**Table 1:** Macroscopic findings observed in 207 reproductive organs from culled sows of the Santa Catarina state, Brazil, with a history of reproductive failure

Macroscopic findings	Farm 1	Farm 2	Farm 3	Total	Percentage (%)
No macroscopic findings	16	115	27	158	76.33
Anestrus	1	9	3	13	6.28
Ovarian follicular cysts	0	8	3	11	5.31
Paratubal cysts	1	4	3	8	3.86
Pregnancy	3	3	2	8	3.86
Fetal maceration	0	3	0	3	1.45
Uterus exudate	0	3	0	3	1.45
Fibrinous inflammation of the perimetrium	0	0	3	3	1.45
Total	21	145	41	207	100.00

**Fig. 2:**
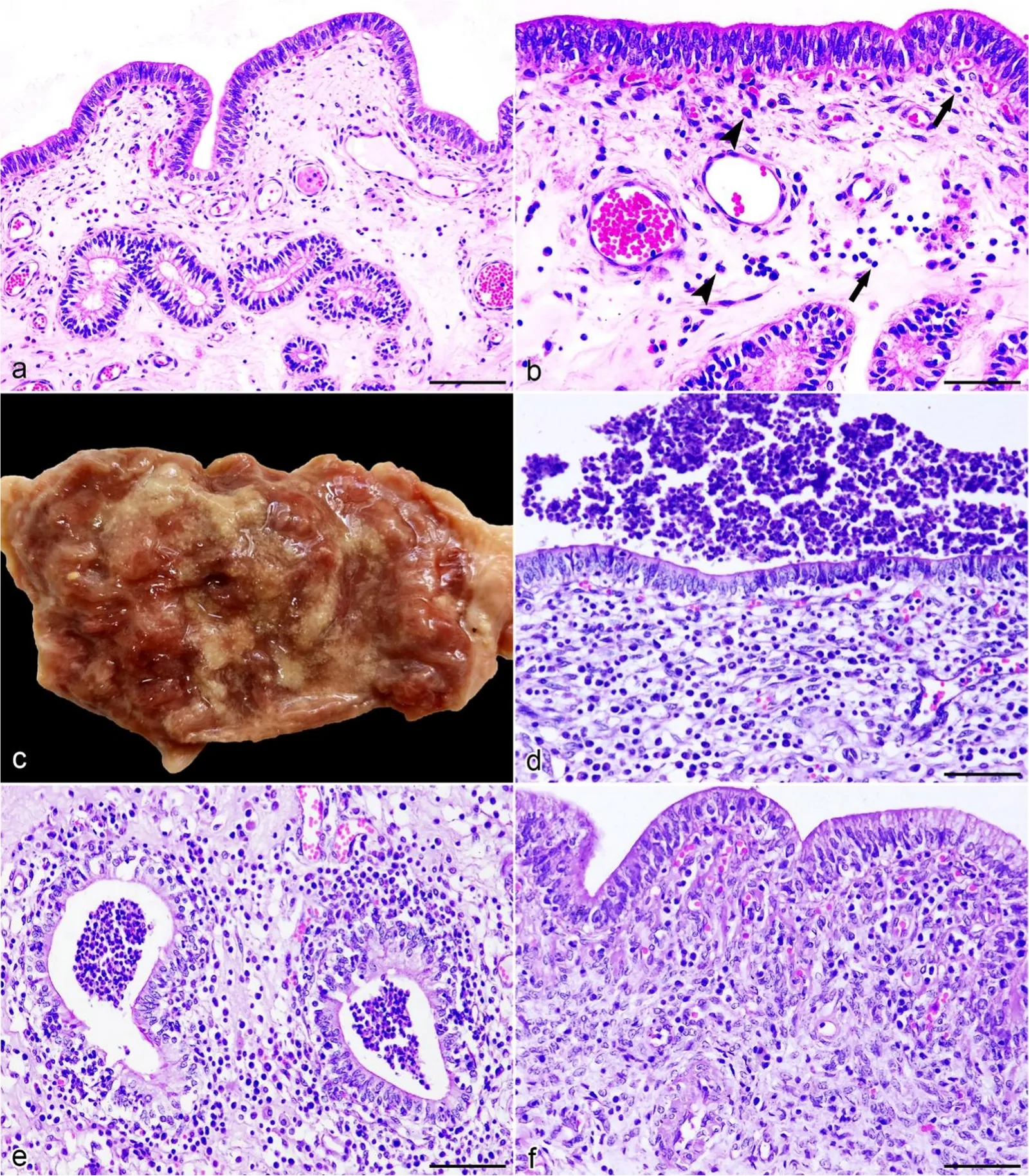
Patterns of endometrial inflammatory findings in sows. (**a)** Endometrium during the follicular phase. Mild subepithelial edema and scattered mild inflammatory cells in the lamina propria. HE, 200x, bar: 100 μm. (**b**) Endometrium during the follicular phase. In more detail, the mild inflammatory cells are composed of mononuclear (arrow) and some polymorphonuclear cells (arrowhead). HE, 400x, bar: 50 μm. **c** Mixed endometritis. Macroscopically, the endometrium shows catarrhal to purulent exudate. (**d)** Mixed endometritis. Microscopically, there are diffuse mixed inflammatory infiltrates in the endometrium in addition to cellular debris in the uterine lumen. HE, 200x, bar: 100 μm. **e** Mixed endometritis. Endometrial glands exhibit intraluminal cellular debris and degenerated neutrophils, which are surrounded by a mixed inflammatory infiltrate. HE, 200x, bar: 100 μm. (**f**) Mononuclear endometritis. The endometrium displays a diffuse inflammatory infiltrate of mononuclear cells. HE, 200x, bar: 100 μm

The histopathological analysis of the uterus revealed the presence of endometritis (21.74%; 45/207). The diagnosed endometrial conditions per farm are shown in Table 2. Most endometritis cases were identified in uteri without evident gross lesions, which explains the discrepancy between gross and histological uterine findings (Tables 1 and 2). In the remaining samples, inflammatory cells in the lamina propria of the endometrium were absent to minimal (78.26%; 162/207) (Fig. 2a-b). Sows classified into estrus (11/162) and probably early diestrus (41/162 - presence of some hemorrhagic corpora lutea) presented more inflammatory cells in the lamina propria of the endometrium (estrus: mean 31 ± 8 inflammatory cells counted; early diestrus: mean 25 ± 10 inflammatory cells counted).

**Table 2:** Endometrial conditions diagnosed from 207 culled sows with a history of reproductive failure originated from three farms in the Santa Catarina state, Brazil

Histopathological pattern	Farm 1	Farm 2	Farm 3	Total	Percentage (%)
Mixed endometritis	2	16	2	20	9.66
Chronic endometritis	2	9	1	12	5.80
Mononuclear endometritis	0	10	0	10	4.83
Sup/fibrinosuppurative endometritis	0	2	0	2	0.97
Pyogranulomatous endometritis	0	1	0	1	0.48
Absent/mild inflammatory cells *	17	107	38	162	78.26
Total	21	145	41	207	100.00

* Criteria refer to inflammatory cells counted within the endometrium

Endometritis lesions were classified into mixed (44.5% [20/45]), chronic (26.7% [12/45]), mononuclear (22.2% [10/45]), suppurative/fibrinosuppurative (4.4% [2/45]), and pyogranulomatous (2.2% [1/45]). Of the 45 samples with endometritis, 57.7% (26/45) presented bacterial growth, and 42.3% (19/45) did not. All isolates obtained were pure cultures exhibiting moderate to high growth. The isolated bacteria included *Escherichia coli* (11 isolates), *Staphylococcus aureus* (9 isolates), *Streptococcus suis* (2 isolates), *Klebsiella* sp., *Corynebacterium* sp., *Trueperella pyogenes*, and *Proteus* sp. (1 isolate each). The agents identified in the lesions are listed in Table 3 and were subdivided according to the histological pattern of the associated endometritis.

**Table 3:** Histopathological patterns of endometrial lesions in sows with a history of reproductive failure and the agents identified through bacteriological and molecular exams

Histopathological patterns	Bacteriological isolation	PCR*
Mixed endometritis (20/207)	*Escherichia coli* (9/20)	PCV2 (2/9)
	*Staphylococcus aureus* (7/20)	PCV2 (1/7); PCV3 (1/7)
	*Corynebacterium* sp. (1/20)	PCV3 (1/1)
	No bacterial growth (3/20)	*Leptospira interrogans* (1/3)
Chronic endometritis (12/207)	*Escherichia coli* (1/12)	Not detected
	No bacterial growth (11/12)	PCV3 (1/11)
Mononuclear endometritis (10/207)	*Staphylococcus aureus* (2/10)	PCV2 (1/2)
	*Escherichia coli* (1/10)	PCV3 (1/1)
	*Klebsiella* sp. (1/10)	Not detected
	*Proteus* sp. (1/10)	Not detected
	No bacterial growth (5/10)	*Erysipelothrix rhusiopathiae* (3/5)
Suppurative/fibrinosuppurative endometritis (2/207)	*Streptococcus suis* (1/2)	Not detected
	*Trueperella pyogenes* (1/2)	Not detected
Pyogranulomatous endometritis (1/207)	*Streptococcus suis* (1/1)	Not detected
Absent to mild endometrium inflammatory cells in the lamina propria (162/207)	No bacterial growth (162/162)	PCV2 (17), PCV3 (9), PPV (14), *Leptospira interrogans* (9), *Erysipelothrix rhusiopathiae* (21)

*Polymerase Chain Reaction represents the detection of the following agents: *Leptospira* sp., *Circovirus porcine2*, *Circovirus porcine3*, porcine parvovirus, *Chlamydia* spp., *Toxoplasma gondii*, *Erysipelothrix rhusiopathiae*, *Brucella suis*, *Listeria* sp., and *Mycoplasma suis*

Grossly, the mixed endometritis pattern did not exhibit significant findings, despite a single sample that presented mild accumulation of catarrhal to purulent exudate in the endometrium (Fig. 2c). Histological examination of all cases, revealed multifocal to diffuse inflammatory infiltrate composed of neutrophils, lymphocytes, plasma cells, and macrophages in the endometrium (Fig. 2d-e). *Escherichia coli* and *Staphylococcus aureus* were the main bacterial agents isolated. Two samples with *E. coli* isolates also showed ovarian anestrus. *Corynebacterium* sp. was identified in a sample, while three samples did not present bacterial growth.

Mononuclear endometritis was characterized by marked, multifocal to diffuse inflammatory infiltrate composed predominantly of lymphocytes, plasma cells, and macrophages (Fig. 2f). Four bacteria were identified: *Staphylococcus aureus*, *Escherichia coli*, *Klebsiella* sp., and *Proteus* sp. No bacterial growth was observed in five samples. Gross lesions were absent in this morphological pattern.

**Fig. 3:**
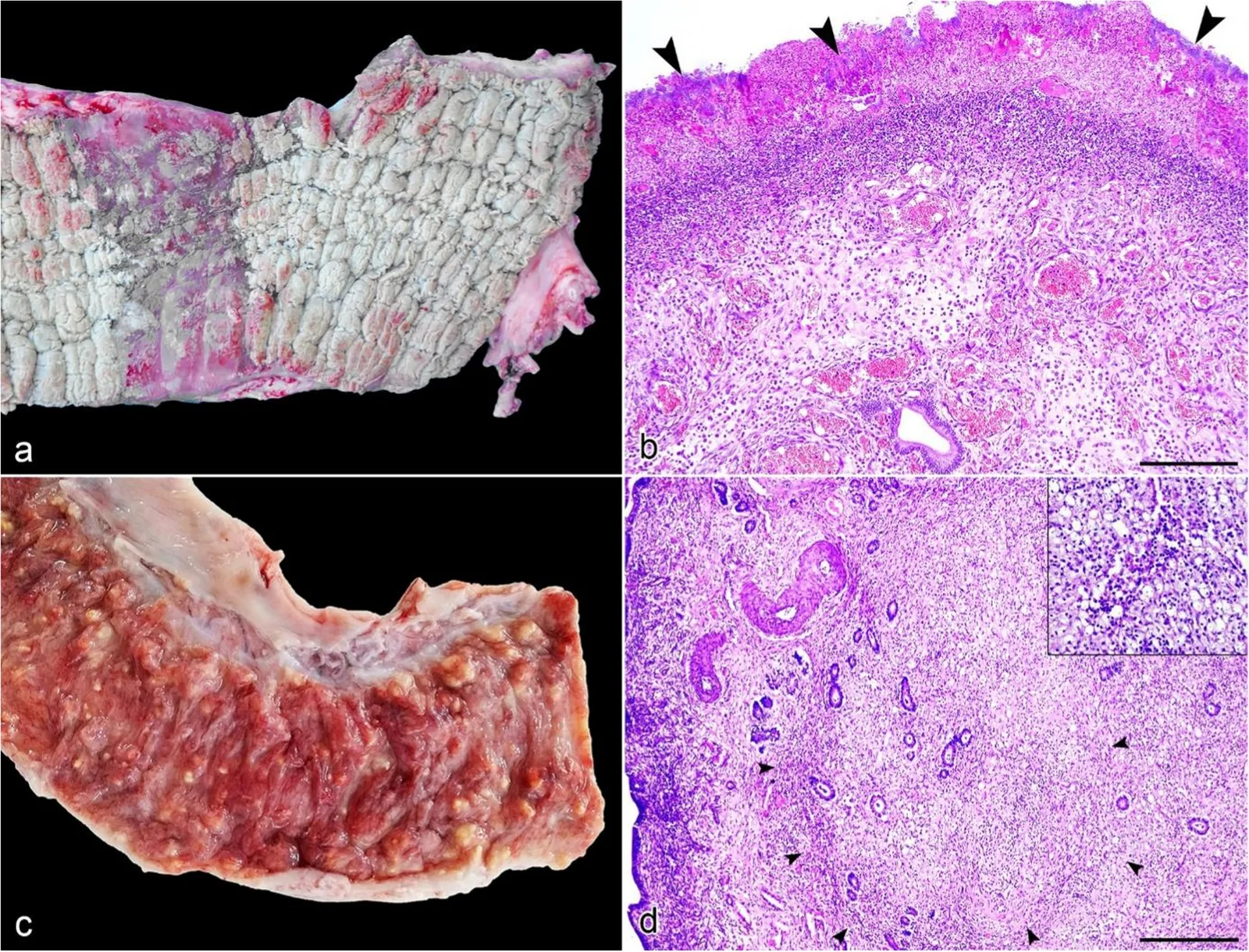
Patterns of endometritis in sows. (**a**) Fibrinosuppurative endometritis. At gross examination, the endometrium contained a marked amount of fibrin deposition and multifocal ulceration. (**b**) Fibrinosuppurative endometritis. Diffuse endometrial ulceration in addition to fibrin deposition. Bacterial aggregates are visualized in the ulcerated areas (arrowhead). HE, 100x, bar: 200 μm. (**c**) Pyogranulomatous endometritis. Multifocal yellow areas, measuring 3–5 mm, slightly arising from the endometrium. (**d**) Pyogranulomatous endometritis. There is a focal area of infiltration by macrophages and degenerated neutrophils in the endometrium (inset), surrounded by delicate connective tissue proliferation (arrowhead). HE, 40x, bar: 500 μm

In the suppurative/fibrinosuppurative endometritis, the endometrium contained a marked amount of fibrin and multifocal ulceration in the macroscopic analysis (Fig. 3a). Histologically, there was a multifocal to diffuse inflammatory infiltrate composed predominantly of degenerated neutrophils, associated with foci of mucosal ulceration and fibrin deposition (Fig. 3b). Bacterial aggregates were occasionally observed in the ulcerated areas of one sample. *Streptococcus suis* and *Trueperella pyogenes* were the pyogenic bacteria identified.

Grossly, pyogranulomatous endometritis presented with slightly yellow, multifocal, areas measuring 3–5 mm, arising from the endometrium (Fig. 3c). Histological analysis revealed the infiltration of macrophages and degenerated neutrophils in the endometrium, sometimes surrounded by mild connective tissue proliferation (Fig. 3d). Although bacteria were not observed in histopathological analysispure growth of *Streptococcus suis* was obtained by culture.

**Fig. 4:**
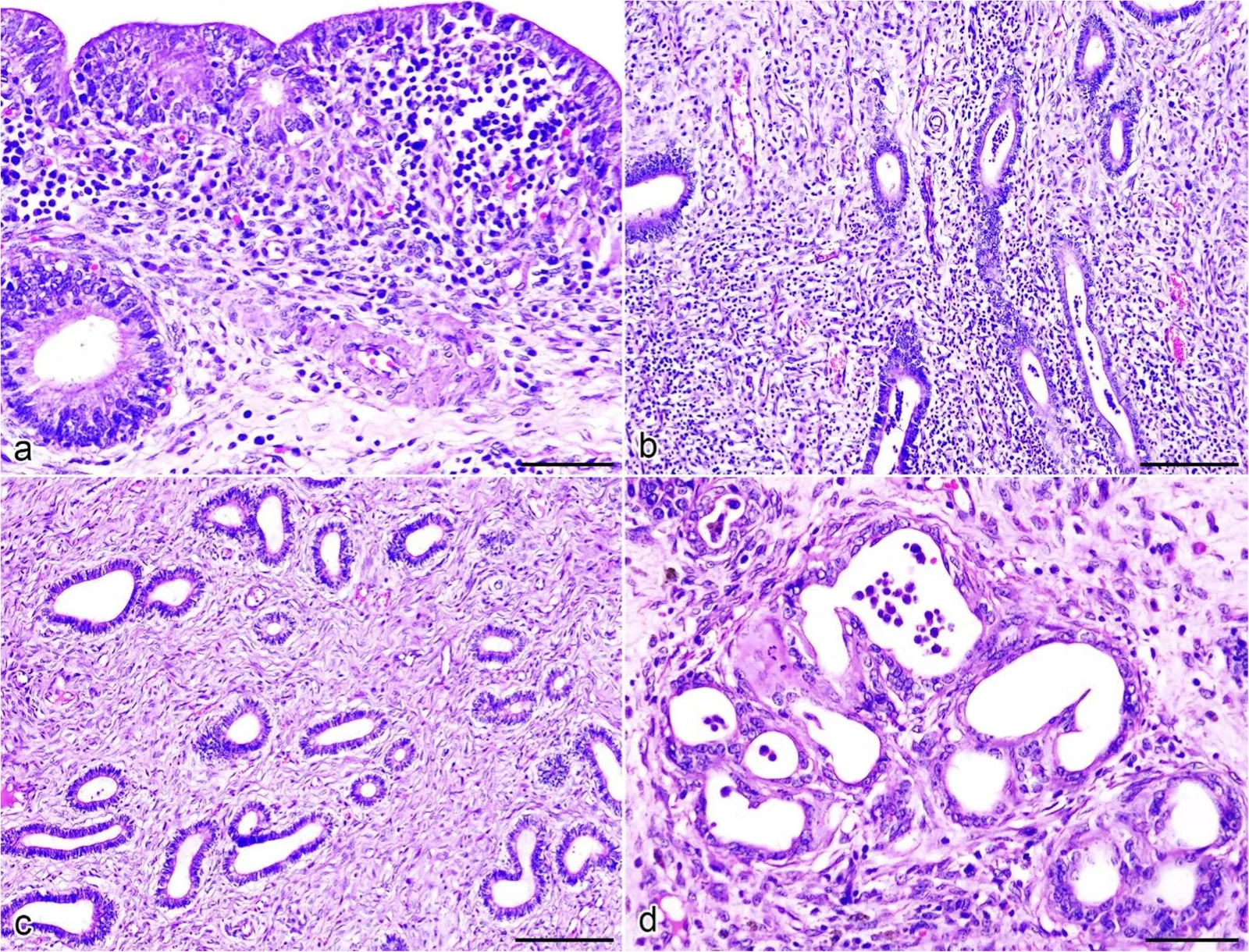
Chronic endometritis in sows. A diffuse mononuclear inflammatory infiltrate in the endometrium with subepithelial lymphocytic aggregates and epithelial attenuation. HE, 200x, bar: 100 μm. (**b**) Endometrial glands surrounded by mononuclear infiltrates and with intraluminal accumulation of cellular debris. HE, 100x, bar: 200 μm. **c** Dilated endometrial glands (glandular ectasia) are spaced and interspersed by connective tissue proliferation. HE, 100x, bar: 200 μm. (**d**) Detail of pronounced glandular ectasia surrounded by fibrous connective tissue (peri-glandular fibrosis). HE, 400x, bar: 50 μm

Chronic endometritis did not evidence macroscopic changes. However, histologic lesions were pronounced and frequently contained endometrial fibrosis in addition to multifocal to diffuse mononuclear inflammatory cells in the endometrium. Occasionally, lymphocytic aggregates in the subepithelial region and multifocal areas of epithelial attenuation were also observed (Fig. 4a-b). Moreover, there was glandular ectasia, with endometrial glands occasionally filled by eosinophilic and amorphous material and surrounded by fibrosis (Fig. 4c-d). Arterial hyalinization was observed in one case.

Regarding the PCR results, twenty-four out of 207 samples (11.6%) were positive for *Erysipelothrix rhusiopathiae*, 10/207 (4.8%) for *Leptospira interrogans*, 21/207 (10.1%) for PCV-2, 13/207 (6.3%) for PCV-3, and 14/207 (6.8%) for PPV. Among these, 12 samples were positive for two agents simultaneously, and three samples were positive for three agents concurrently (Suppl. Table). These detections occurred in samples either with or without histological evidence of endometritis (Table 3). *Toxoplasma gondii*, *Chlamydia* spp., *Brucella suis*, *Listeria* sp., and *Mycoplasma suis* were not detected in this study.

## Discussion

Reproductive failure associated with abortions in swine herds is extensively studied worldwide, particularly in Brazil. Research emphasizes infectious causes, especially of viral origin, such as *Circovirus porcine2* and *Circovirus porcine3*, porcine parvovirus, and porcine reproductive and respiratory syndrome virus (PRRSV), although PRRSV has not yet been diagnosed in Brazil. In addition to viral pathogens, several bacterial and parasitic agents, such as *Leptospira* spp., *Brucella* spp., *Erysipelothrix rhusiopathiae*, *Chlamydia* spp., and *Toxoplasma gondii*, are well-recognized contributors to swine reproductive disorders (Althouse et al. 2019; Maes et al. 2023; Pozzi and Alborali 2012). The identification of the etiological agent typically relies on samples collected shortly after abortion, including placental tissues, aborted fetuses, or maternal serum, with diagnostic approaches aimed at detecting the pathogen during the acute phase of infection (Pescador et al. 2010; Althouse et al. 2019). In the present study, we investigated the major viral and bacterial agents associated with reproductive failure using PCR analysis of uterine mucosa collected at slaughter.

Molecular screening revealed several reproductive pathogens in uterine samples, including *Erysipelothrix rhusiopathiae* (11.6%), *Leptospira interrogans* (4.8%), PCV-2 (10.1%), PCV-3 (6.3%), and PPV (6.8%). The findings provide important epidemiological data, indicating the circulation of these agents within the evaluated herds. However, PCR-positive results alone do not confirm reproductive failure by these agents. Also, most PCR-positive detections occurred in samples lacking histological evidence of endometritis and had PCR Ct values > 30. These findings suggest that molecular positivity primarily reflects pathogen circulation rather than an active etiological role in the uterine samples. Consequently, PCR findings should be interpreted cautiously in conjunction with bacteriological and histopathological examinations, emphasizing the importance of integrated diagnostic approaches for investigating reproductive failure in swine.

On the other hand, PCR is a very sensitive tool; thus, positive results may reflect prior exposure, transient colonization, or residual DNA persistence after immune clearance, particularly under subclinical or chronic conditions characterized by low bacterial or viral loads. Notably, the discrepancy between PCR positivity and the absence of bacterial isolation, as observed for *E. rhusiopathiae*, is not unexpected. This organism is fastidious, exhibits slow growth, and has limited competitiveness in mixed microbial cultures, which markedly reduces its recovery from uterine samples. Also, the use of abattoir-derived samples represents an additional limitation, as these are typically collected outside the acute phase of infection, further compromising the bacterial isolation.

The identification of viral agents such as PCV-2, PCV-3, and PPV, pathogens known for their ability to persist in tissues, should also be interpreted with caution, even in cases of endometritis, as detection does not necessarily indicate clinical significance or a direct contribution to reproductive inefficiency. In a study with culled gilts, Pearodwong et al. (2015) detected PCV-2 DNA in 50% of uterine samples without lesions and in 40% of samples with endometritis. Similar results were observed by Limsaranrom et al. (2016), who found PCV2 DNA in both normal uteri (7/13, 53.9%) and in the uteri with acute endometritis (4/11, 36.4%). Malgarin et al. (2019) also detected PRRS in the endometrium 14 days after infection.

Bacteria identified, such as *Escherichia coli*, *Streptococcus suis*, *Trueperella pyogenes*, *Corynebacterium* sp., and *Staphylococcus aureus*, are well-known causes of abortion in swine (Althouse et al. 2019; Maes et al. 2023; Pescador et al. 2010; Pozzi and Alboralli 2012). Bacterial infection is the main cause of endometritis in sows, and the agents are reportedly associated with reproductive failure in swine (Althouse et al. 2019; de Jong et al. 2014). In a large-scale study conducted in Italy with 140 farms, infectious agents were identified in 323 out of 549 abortion cases, with *E. coli* (64/323), *Streptococcus* spp. (63/323), and *Staphylococcus* spp. (5/323) being the most commonly isolated (Salogni et al. 2016). These bacteria can induce abortion in swine, primarily through ascending infections via the open cervix, and less commonly via hematogenous spread, leading to placental infection. Our findings further support that these bacterial species can also be recovered from uterine samples of culled sows at slaughter, highlighting their potential role in subclinical or undiagnosed reproductive disorders.

Endometritis is the most common lesion found in culled sows, especially in those culled due to reproductive failure (de Jong et al. 2014). Inflammatory endometrial lesions may be detectable macroscopically by evaluation of the uterine mucosa in severe cases; however, endometritis is not invariably identifiable clinically within herds or may not always be apparent grossly in uterine samples from culled sows. To confirm the diagnosis of endometritis, histopathological examination is essential, mainly in chronic cases. Such chronic endometrial lesions are well characterized and established in mares, with reports also existing in cows and bitches (Foster and Premanandan 2022; Barros and Masuda 2013; Gifford et al. 2014; de Witt et al. 2023). Severe cases of chronic endometritis may result in early embryonic loss, abortion, or an inability to sustain pregnancy to term without specific gross changes (Foster and Premanandan 2022). In sows and gilts, investigations have documented chronic endometrial lesions associated with reproductive failures, often linked to bacterial infections (Dalin et al. 2004; Tummaruk et al. 2010; de Jong et al. 2014). Reports have indicated that 81% of slaughtered sows examined exhibited chronic uterine lesions, highlighting a significant association between chronic inflammation and reproductive dysfunction (de Jong et al. 2014). In our study, the prevalence of chronic endometritis was comparatively lower (12.4%). Nonetheless, we conducted a systematic and careful analysis of the chronic lesions based on established histopathological criteria known to significantly reduce the likelihood of carrying a pregnancy to term in mares (Snider et al. 2011; de Witt et al. 2023). The morphological features of the chronic alterations observed in sow endometria in this investigation closely resemble those described in mares, suggesting a correlation between reproductive failure and these lesions. However, caution must be exercised when comparing domestic species, and additional clinical parameters should be incorporated to definitively determine sow infertility, as has been established in the context of mare infertility.

Another critical aspect to avoid misdiagnosis of endometritis or reproductive failure from uterus samples is the interpretation of the inflammatory cells in the endometrium. As the sampled sows in our study were culled for non-specific reasons, we possibly collected many samples from sows with no reproductive failure (as shown by the high number of diestrus detected – cycling sows). During the follicular phase, inflammatory cells, including neutrophils, lymphocytes, plasma cells, and macrophages, can be found in the lamina propria of the endometrium, mainly in the subepithelial layers, while they decrease significantly during diestrus, especially in late diestrus (Blackwell et al. 2003; Kaeoket et al. 2002; Dalin et al. 2004; Tummaruk et al. 2010). The resident inflammatory cell thresholds within the endometrium (Dalin et al. 2004; Tummaruk et al. 2010) and the histological patterns of endometritis described in our study may aid in differentiating pathological changes from physiological processes.

The integration of bacteriological, histopathological, and molecular examinations represents a practical approach for investigating the reproductive tract of culled sows (Dalin et al. 1997; Tummaruk et al. 2009; de Jong et al. 2014). The evaluation of a large number of uterine samples in this study enabled the characterization of histological patterns of endometritis, which may assist pathologists in correlating microscopic lesions with bacteriological findings, as significant bacterial growth was primarily observed in cases with histological evidence of endometritis. In contrast, PCR findings were not consistently associated with histological evidence of endometritis and therefore suggest a weak relationship with the microscopic lesions. Nevertheless, negative PCR results do not exclude prior infection, as pathogen DNA may be absent in chronic or resolved cases. Overall, the lack of evidence of pathogen localization within lesions limits causal inference, and techniques enabling in situ detection would be required to clarify etiological relationships.

A high number of lesions related to reproductive failures have been described by other studies conducted on reproductive organs from culled sows (Dalin et al. 1997; Heinonen et al. 1998; Tummaruk et al. 2009; Ritterbusch et al. 2012; de Jong et al. 2014; Pearodwong et al. 2015; Limsaranrom et al. 2016; Egli et al. 2025). In this context, sampling of slaughtered sows can still serve as a valuable herd-level screening and complementary tool for herd-level surveillance, enabling the detection of potential causes associated with reproductive failure within the herds. Additionally, as slaughterhouses receive animals from multiple production stages and varying reproductive outcomes, these samples provide a representative overview of herd health and can assist in recognizing patterns that may be overlooked during routine farm monitoring.

Ovarian lesions, especially ovarian cysts, are common reproductive abnormalities identified in swine, as well as in other domestic species such as cattle and mares. However, considerable variation exists among different reports. In our findings, 5.31% of ovaries exhibited follicular cysts. A recent study reported that approximately 10% of culled sows in Switzerland had single or multiple ovarian cysts (Egli et al. 2025), whereas percentages in Finland and Brazil were 6.2% (Heinonen et al. 1998) and 2.4% (Castagna et al. 2004), respectively. These cystic structures can disrupt normal ovarian function and are frequently linked to hormonal imbalances, particularly involving luteinizing hormone, leading to ovulation failure, anestrus, and prolongation of the postpartum interval until the first estrous cycle (Castagna et al. 2004; de Jong et al. 2014; Foster and Premanandan 2022). The presence of ovarian cysts may also be correlated with a shortened lactation period and reduced weaning-to-estrus intervals. However, such an association was not corroborated by our results, as we lacked recorded reproductive histories for the sampled sows, although previous studies have demonstrated this through various methodological analyses (Castagna et al. 2004). Bacterial endometritis, particularly caused by *E. coli* infections, may also be associated with the development of anovulatory follicles and anestrus (Foster and Premanandan 2022; Jana et al. 2004), a finding correlation observed in only two of our samples exhibiting both endometritis and anestrus. Conversely, paratubal cysts, accounting for 3.86% of our samples, are common findings in sows yet are rarely linked to reproductive failure (Heinonen et al. 1998).

Fetal maceration occurs when one or more fetuses are not expelled during parturition, resulting in death and liquefaction of fetal tissues. The retained fetus favors bacterial growth, predisposing the sow to postpartum endometritis. If not resolved, the localized infection can progress to sepsis, which may be fatal in severe cases. While sows with this condition may still exhibit signs of estrus, the persistent uterine infection and associated tissue damage typically impair the ability to establish or maintain a subsequent pregnancy (Foster and Premanandan 2022). On the other hand, pregnant culled sows were identified on all three farms, which may reflect misdiagnosis of reproductive status by farm staff. However, because no prior individual clinical histories were available, these animals may also have been culled for non-reproductive reasons, such as locomotor disorders (de Jong et al. 2014).

Another lesion observed was fibrinous inflammation of the perimetrium, identified in sows with bacterial peritonitis or abscedative lesions at slaughter, all from farm 3. The primary cause of these lesions was not determined, and no bacteria were isolated. Notably, the endometrium in these cases did not exhibit inflammatory changes. Peritonitis in adult pigs is uncommon; however, *Streptococcus equi* subsp. *zooepidemicus* and other pyogenic bacteria should be considered as possible etiological agents (Sitthicharoenchai et al. 2020). Although not a primary reproductive tract pathology, systemic lesions such as peritonitis may indirectly contribute to reproductive failure and abortion through mechanisms such as endotoxemia and fever (Althouse et al. 2019; Foster and Premanandan 2022). Therefore, these findings should be interpreted in the context of clinical history, as reproductive failure may reflect an underlying systemic condition rather than a primary reproductive disorder.

A limitation of this study is the lack of individual sow data from the pig farms analyzed, which precluded investigation into specific reproductive histories or individual-level factors linked to reproductive failure. Also, sampling at slaughterhouses may introduce bias related to age, parity, and herd selection, as culled sows may not be fully representative of the broader breeding population. Furthermore, although the detection of pathogen DNA by PCR provides valuable epidemiological information, it does not imply causation, nor does it imply the occurrence of latent or transient infections that may not be directly associated with reproductive disorders. The timing of sampling at slaughter may also limit interpretation, as it does not necessarily coincide with critical reproductive events or periods of acute infection. Moreover, detection of the agents identified by qPCR within microscopic lesions would strengthen the biological relevance of the results. Nevertheless, slaughterhouse-based screening can be a useful tool when integrated into herd health programs, guiding future diagnostic approaches and identifying potential pathogens circulating within herds. A more comprehensive diagnostic strategy, including fetal tissues, placental samples, and maternal serum collected during or shortly after abortion, remains critical for increasing the likelihood of identifying causative agents in clinical cases from the analyzed farms.

In conclusion, this study highlights that reproductive tract evaluation at slaughter can identify uterine and ovarian lesions in sows from farms with low reproductive performance indices. Endometritis was the main lesion identified at slaughter, with *E. coli*, *Staphylococcus aureus*, and *Streptococcus suis* being the most frequently isolated bacteria. Molecular screening also revealed the circulation of important reproductive pathogens, including PCV-2, PCV-3, PPV, *Leptospira* spp., and *Erysipelothrix rhusiopathiae*. Histopathological analysis revealed that chronic endometritis lesions shared features with those described in other domestic species, especially mares, which might impact fertility. Nevertheless, further histopathological and clinical research is warranted to better understand these chronic lesions and PCR-detected agents in uterine samples, as well as their potential role in swine reproductive disorders, particularly given the lack of individual reproductive histories of the sampled sows. Overall, the findings also support the inclusion of reproductive tract analysis at slaughter in herd health monitoring programs and underscore the need for further studies linking histological findings with reproductive performance and clinical outcomes.

## Supplementary Information

Below is the link to the electronic supplementary material.

Supplementary Material 1 (XLSX 17.9 KB)

## Data Availability

All data from this manuscript are included in this article’s version.
